# Delphi Consensus on the Use of Fenofibrate as Systemic Therapy for the Prevention of Diabetic Retinopathy Progression

**DOI:** 10.1111/1753-0407.70193

**Published:** 2026-02-12

**Authors:** Richard C. O'Brien, Khalifa Abdallah, Xuehong Dong, Mohamed Hassanein, Qiuhe Ji, Sakir Ozgur Keskek, Patricio Lopez‐Jaramillo, Roopa Mehta, Hussein Raef, Kevin Tan

**Affiliations:** ^1^ Austin Clinical School, Melbourne Medical School University of Melbourne Melbourne Australia; ^2^ Diabetes and Metabolism Unit, Department of Internal Medicine Alexandria University Alexandria Egypt; ^3^ Department of Endocrinology and Metabolism Sir Run Run Shaw Hospital of Zhejiang University Hangzhou China; ^4^ Department of Endocrinology and Diabetes Dubai Hospital, Dubai Academic Health Cooperation Dubai UAE; ^5^ Department of Endocrinology and Metabolism Xi'an International Medical Center Hospital of Northwest University Xi'an Shaanxi China; ^6^ Department of Internal Medicine, School of Medicine Alanya Alaaddin Keykubat University Antalya Turkey; ^7^ Masira Research Institute, Medical School, Universidad de Santander Bucaramanga Colombia; ^8^ Departamento de Endocrinología y Metabolismo and Unidad de Investigación en Enfermedades Metabólicas (UIEM) Instituto Nacional de Ciencias Médicas y Nutrición Salvador Zubirán México City México; ^9^ Department of Executive Health Medicine King Faisal Specialist Hospital and Research Center Riyadh Saudi Arabia; ^10^ Farrer Park Hospital Singapore Singapore

**Keywords:** consensus, diabetic retinopathy, fenofibrate

## Abstract

**Background:**

Diabetic retinopathy remains a leading cause of preventable blindness worldwide, yet screening and management practices vary widely. Evidence suggests that systemic therapies, including fenofibrate, may slow diabetic retinopathy progression, but their use is inconsistent across clinical settings. This study aimed to establish an evidence‐informed consensus among endocrinology experts on the screening, diagnosis, and treatment of diabetic retinopathy, with a particular focus on recommendations for the use of systemic therapy to prevent disease progression.

**Methods:**

A modified three‐round Delphi process was conducted with 19 endocrinology experts from diverse geographic regions. A core panel of 10 experts and an extended panel of 9 reviewed and rated 19 evidence‐based statements. Consensus was defined as > 75% agreement.

**Results:**

All 19 statements achieved consensus, with 14 receiving > 80% agreement. The panel endorsed frequent diabetic retinopathy screening based on diabetes type and risk level, early initiation of fenofibrate in patients with mild to moderate non‐proliferative diabetic retinopathy, and continued therapy to sustain retinal protection. Fenofibrate was recognized for its pleiotropic effects, and the experts agreed that the transient rise in serum creatinine with fenofibrate is not indicative of renal damage and should not prompt discontinuation.

**Conclusions:**

This consensus highlights the need for multidisciplinary care, coordinated pathways, and patient education in diabetic retinopathy care. It also offers unified, evidence‐informed recommendations for endocrinologists for the early initiation of fenofibrate to reduce diabetic retinopathy progression. While further studies are needed, these findings offer a practical framework for improving diabetic retinopathy management globally.

## Highlights

- Nineteen endocrinology experts from nine countries participated in a Delphi consensus on systemic therapy for diabetic retinopathy.
- Consensus supported early and sustained use of fenofibrate to slow retinopathy progression and reduce invasive interventions.
- Experts emphasized coordinated multidisciplinary care and patient education to optimize outcomes globally.

## Graphical abstract

Nineteen international endocrinologists support fenofibrate use to prevent diabetic retinopathy progression and emphasize the need for coordinated care pathways and early intervention to improve diabetic retinopathy management globally.

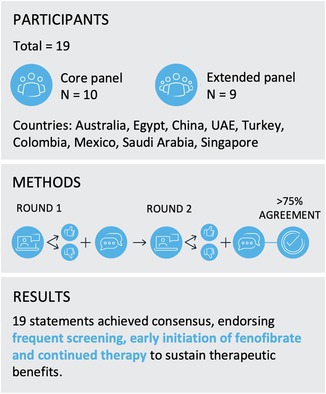

## 1 Introduction

Diabetic retinopathy is a common complication of diabetes mellitus and remains a leading cause of preventable vision loss globally [1]. As the prevalence of diabetes continues to rise, reaching an estimated 537 million people worldwide in 2021 and projected to exceed 700 million by 2045, the burden of diabetic retinopathy has grown in parallel [2]. Among people living with diabetes, approximately one in three will develop some degree of diabetic retinopathy, and about one in three of these individuals will go on to develop vision‐threatening diabetic retinopathy, such as proliferative diabetic retinopathy (PDR) or diabetic macular edema [3]. Without timely identification and intervention, these advanced stages can lead to serious complications, including vitreous hemorrhage, tractional retinal detachment, and neovascular glaucoma, ultimately resulting in irreversible visual impairment or blindness [4].

The disease burden is particularly profound among individuals with longstanding diabetes. More than 75% of people with Type 2 diabetes will develop some form of diabetic retinopathy 20 years after diagnosis [3]. Key risk factors for the development and progression of diabetic retinopathy include the duration of diabetes, suboptimal glycemic control, elevated blood pressure, and abnormal lipid profiles [3]. Importantly, diabetic retinopathy is typically asymptomatic in its early stages, underscoring the necessity for regular, comprehensive ophthalmic screening in all individuals with diabetes [5]. Early detection and management are critical to halting disease progression and preserving vision.

Current management of early‐stage, non‐proliferative diabetic retinopathy (NPDR) focuses on stringent control of modifiable systemic risk factors, including glycemic, blood pressure, and lipid management [5]. In recent years, attention has also turned to the potential role of systemic pharmacologic agents in delaying or preventing the progression of diabetic retinopathy. One such agent, fenofibrate, has demonstrated efficacy in large clinical trials such as FIELD, ACCORD‐Eye, and LENS, showing reduced progression of diabetic retinopathy in patients with Type 2 diabetes [6, 7, 8, 9]. However, despite this growing body of evidence, the adoption of systemic therapy in diabetic retinopathy management varies widely in clinical practice.

A significant challenge in optimizing diabetic retinopathy care lies in the lack of global consensus on screening protocols, preventive strategies, and treatment pathways, particularly regarding the role of systemic therapy. The patient journey for individuals with diabetic retinopathy is often inconsistent, with variability in screening access, referral processes, disease monitoring, and treatment delivery. This heterogeneity can delay interventions and compromise outcomes. Additionally, although most major guidelines acknowledge the role of fenofibrate in slowing the progression of existing diabetic retinopathy, they notably lack specific recommendations regarding treatment administration, such as the optimal timing for initiation and the appropriate duration of treatment [5, 10, 11, 12].

There is a pressing need for a unified, evidence‐informed approach to guide clinicians and healthcare systems in the effective prevention and management of diabetic retinopathy. To address these gaps and harmonize care across regions, we conducted a Delphi consensus process involving a diverse panel of leading experts in endocrinology. Our objective was to develop consensus‐based recommendations for endocrinologists focused on the use of fenofibrate for the prevention of diabetic retinopathy progression. By integrating multidisciplinary perspectives and aligning on evidence‐based strategies, this initiative seeks to inform clinical practice, support guideline development, and ultimately improve outcomes for people with diabetes worldwide.

## 2 Methods

### 2.1 Consensus Participants

The Delphi consensus involved a core panel, comprised of one Scientific Coordinator (R. C. O'B.) and nine additional experts. Panelists with practical experience in the use of fenofibrate to treat diabetic retinopathy were selected to represent seven countries where fenofibrate is approved for this indication (Australia, Egypt, China, Turkey, Colombia, Mexico, and Singapore). To broaden the relevance of the consensus, experts from two additional countries (United Arab Emirates [UAE] and Saudi Arabia), which face a high burden of diabetic retinopathy but lack regulatory approval for fenofibrate in this indication, were also included to provide complementary perspectives. Panel members were selected based on predefined criteria, including relevant peer‐reviewed publications, recognized clinical expertise, and significant contributions in the field of diabetic retinopathy. The members of the core panel suggested up to two additional experts (nine total) to form the extended panel, who participated in the second round (Table 1).

**TABLE 1:** Participants involved in the Delphi consensus.

Name	Country	Round 1	Round 2	Round 3
**Core panel**				
Richard O'Brien *(Scientific Coordinator)*	Australia	X	X	X
Khalifa Abdallah	Egypt	X	X	X
Xuehong Dong	China	X	X	X
Mohamed Hassanein	UAE	X	X	X
Qiuhe Ji	China	X	X	X
Sakir Ozgur Keskek	Turkey	X	X	X
Patricio Lopez‐Jaramillo	Colombia	X	X	X
Roopa Mehta	Mexico	X	X	X
Hussein Raef	Saudi Arabia	X	X	X
Kevin Tan	Singapore	X	X	X
**Extended panel**				
Basel Al Sarraj	Saudi Arabia	—	X	—
Gao Bin	China	—	X	—
Guillermo Gonzalez Galvez	Mexico	—	X	—
Husam Jnaid	Saudi Arabia	—	X	—
Chung Horn Lee	Singapore	—	X	—
Fawzy Messallamy	Egypt	—	X	—
Semir Paşa	Turkey	—	X	—
Jiayue Wang	China	—	X	—
Yang Yan	China	—	X	—

### 2.2 The Consensus Process

An independent facilitator (Circular Communications, UK) developed the initial statements with supporting references based on a comprehensive review of the latest scientific literature and guidelines. The statements were then reviewed and refined by the Scientific Coordinator.

**FIGURE 1:**
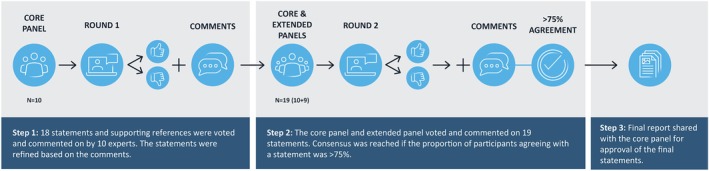
The Delphi consensus process.

The Delphi procedure involved three rounds (Figure 1). Each round was conducted via an online platform developed by an external partner (WeLoveDigi, Bulgaria). The platform facilitated the assembly of the Delphi panel and the conduction of the consensus process in a predefined, standardized manner, irrespective of time zone or geographic location of the panelists.

Round 1: The core panel (*n* = 10) reviewed the initial statements and indicated their level of agreement using a five‐point Likert scale: Strongly Agree, Agree, Neutral, Disagree, Strongly Disagree. Panelists could also provide written comments to justify their responses or suggest revisions. If the core panelists requested changes to a statement's wording or content, the statement was revised accordingly. All votes and comments were compiled into a preliminary summary report. This report was shared with the core panel as preparatory reading for Round 2.

Round 2: The second round followed the same voting format, now including both the core panel and the extended panel (*n* = 19). Statements were revised according to panelist comments and statements reaching > 75% agreement were considered as agreed.

Round 3: A final summary report was shared with the core panel, summarizing the results from Round 2. All core panelists reviewed and formally approved the final statements.

### 2.3 Role of the Sponsor

Funding was provided by Abbott Products Operations AG, Allschwil, Switzerland for a third‐party scientific communications agency (Circular Communications, UK) to assist the scientific coordinator (R.C.O'B.) to formulate the initial statements, facilitate the Delphi consensus process and support manuscript preparation. The voting results were collected via a Delphi platform developed and managed by a third‐party agency (WeLoveDigi, Bulgaria). Abbott Products Operations provided funding for the development of the Delphi platform. The sponsor played no part in the Delphi process or the manuscript preparation.

## 3 Results

### 3.1 The Delphi Process

Eighteen statements were developed and approved by the scientific coordinator to be included in the Delphi consensus. They were categorized into four topics: Screening & Diagnosis, Treatment & Prevention of Progression, Treatment Duration, and Fenofibrate & Kidney Function.

In Round 1, sixteen out of the eighteen proposed statements achieved > 75% agreement by the core panel (Supplementary Table 1). Amendments were made to thirteen of the statements, and one new statement was added (statement 2.7) based on the panelists' comments. In Round 2, all nineteen statements achieved > 75% agreement by the core panel and extended panel (Table 2). Three statements achieved > 90% agreement, and eleven achieved > 80% agreement. Fifteen of the statements were amended based on the panelists' comments. The final report, containing a summary of the voting and the final statements, was approved by each member of the core panel.

**TABLE 2:** Consensus statements and Round 2 agreement level.

Statement number	Statement content	Agreement level
1.1	Adults with Type 1 diabetes should be offered annual eye screenings beginning 5 years after diagnosis. Adults with type 2 diabetes should be offered a prompt eye screening at the time of diagnosis. Thereafter, people with no signs of retinopathy can be offered screening every 1–2 years, while those with signs of retinopathy should have more frequent screening determined based on the severity of their condition. Additionally, patients with uncontrolled Type 2 diabetes should be screened more frequently due to the risk posed by hyperglycemia as well as rapid HbA1c reduction which may cause transient worsening or retinopathy. The aim is to identify signs of diabetic retinopathy and facilitate earlier intervention before the disease progresses to be vision threatening. Patients should be counseled on the importance of attending regular screening appointments, even if they are asymptomatic, to reduce their risk of diabetes‐associated vision impairment	92.11%
1.2	Screening for diabetic retinopathy should utilize retinal photography, preferably digital, or dilated eye examination as primary methods. Retinal photography can be performed in the primary care setting by a trained healthcare provider. Dilated eye examinations should be conducted by an optometrist or ophthalmologist	86.84%
1.3	Any patient identified with evidence of moderate or worse non‐proliferative diabetic retinopathy, proliferative diabetic retinopathy or significant changes compared to their previous examination should be promptly referred to an ophthalmologist for further management	93.42%
1.4	Early identification and subsequent systemic treatment of diabetic retinopathy play a critical role in reducing the risk of progression to proliferative retinopathy and avoiding costly, invasive, and high‐risk treatments for advanced disease, such as retinal laser and intravitreal injections	93.42%
1.5	Achievement of an individualized HbA1c target should be emphasized as a critical measure to prevent the progression of diabetic retinopathy, alongside management of other systemic risk factors like hypertension and lipid levels. An HbA1c target of < 7% is reasonable for most adults, but the target should be personalized based on disease duration, life expectancy, use of pharmacological agents, comorbidities, BMI, genetic predisposition, and established vascular complications, while also ensuring a low risk of hypoglycemia. When HbA1c is high (~ > 8.5%), a gradual decrease is advised, especially when using GLP‐1 agonists, insulin and GIP‐GLP1 co‐agonists, to avoid transient worsening of diabetic retinopathy associated with intensive glycemia lowering	85.53%
1.6	Effective screening for diabetic retinopathy requires a systematized screening pathway to be in place in order for patients to receive preventative treatment in a timely manner. This should ideally include: A diabetes register to ensure accurate patient tracking, with flexible methods for call and recall to invite patients for screening. Electronic Health Record‐based tracking systems, where feasible, to send automated invitations and reminders and to flag patients who are overdue for follow‐up appointments. Streamlined referral pathways, with direct electronic communication between primary care providers and specialists, prompt follow‐up, and clear guidance for patients on the next steps. The use of telemedicine to maintain regular contact with patients, especially those in rural or underserved areas, to reduce the likelihood of missed appointments or delays in follow‐up. Public service announcements to raise awareness about the importance of screening and early detection. Mobile testing units in underserved areas to reduce travel barriers and address inequitable access.	88.16%
2.1	Most treatments that have conventionally been used to treat diabetic retinopathy are used to help halt disease progression or reduce vision loss at fairly advanced stages and are invasive. These treatments can be complex, expensive, involve multi‐stage methods, and require repeated administration. Therefore, systemic treatment options that are minimally invasive and can be implemented long‐term are needed to prevent disease progression at the early stages and help preserve vision	86.84%
2.2	Fenofibrate shows promise as an effective therapy to slow the progression of diabetic retinopathy and reduce the need for more expensive and invasive treatments such as laser therapy, in combination with management of other risk factors, particularly in patients with Type 2 diabetes.	78.95%
2.3	Physicians should consider fenofibrate treatment at the first signs of mild or moderate non‐proliferative retinopathy, regardless of blood glucose control or triglyceride levels, to reduce the risk of progression to proliferative diabetic retinopathy (PDR). Optimal management of other risk factors is also needed. Conversations with patients should include a clear explanation of fenofibrate's role in retinopathy management and its independence from lipid levels.	78.95%
2.4	Treatment with statin medications, which are primarily used to manage dyslipidemia, reduce LDL cholesterol, and halt cardiovascular disease, does not appear to significantly alter the course of diabetic retinopathy.	85.53%
2.5	Fenofibrate has been shown to reduce the progression of existing diabetic retinopathy when used as monotherapy and also in combination with a statin.	85.53%
2.6	Fenofibrate treatment is particularly beneficial for patients with pre‐existing diabetic retinopathy. Studies show that its use provides significant protection against further retinal damage, particularly in those already exhibiting early signs of the disease.	89.47%
2.7	Fenofibrate may exert protective effects on the retina through several molecular mechanisms, including its anti‐inflammatory, anti‐apoptotic, anti‐angiogenic, and antioxidant properties. By activating peroxisome proliferator‐activated receptor‐alpha (PPAR‐*α*), fenofibrate reduces the expression of pro‐inflammatory cytokines and vascular endothelial growth factor (VEGF), which are implicated in the progression of diabetic retinopathy. Additionally, fenofibrate decreases oxidative stress by inducing the expression of antioxidant enzymes, reducing the production of reactive oxygen species, contributing to its ability to protect retinal cells and maintain vascular integrity.	78.95%
3.1	Fenofibrate treatment should be considered long‐term for patients with non‐proliferative and proliferative diabetic retinopathy as it may slow disease progression and protect against vision‐threatening complications. Randomized trials with a median duration of 4.0 years have shown a reduction of progression of diabetic retinopathy in patients treated with fenofibrate compared to placebo. More long‐term studies are needed to confirm the long‐term effects.	81.58%
3.2	Long‐term treatment with fenofibrate can reduce the need for more invasive ophthalmologic interventions, such as laser therapy, in adults with Type 2 diabetes. More studies which involve type 1 diabetes and assess other ophthalmologic interventions are needed.	80.26%
3.3	The therapeutic benefits of fenofibrate are lost once treatment is discontinued, and therefore sustained use is crucial to maintain its protective effects.	84.21%
4.1	When fenofibrate is initiated, a mild and reversible increase in serum creatinine levels is commonly observed, up to 30%, typically within the first few weeks of treatment. After the initial rise, creatinine levels usually stabilize and remain elevated but do not increase further throughout the duration of fenofibrate treatment. Upon discontinuation of fenofibrate, creatinine levels typically return to baseline or near‐baseline values.	82.89%
4.2	The mechanism behind the increase in serum creatinine levels is not yet fully understood, but it appears to involve multiple factors. The elevation may result from reduced creatinine clearance despite a preserved inulin‐derived glomerular filtration rate (GFR), suggesting that the active secretion pathway of creatinine in the proximal tube may be impaired rather than glomerular function. Altered renal hemodynamics may also play a causative role. Another potential contributor is an increase in creatinine production by muscle.	77.63%
4.3	In two randomized controlled trials, treatment with fenofibrate was shown to slow the progression of renal function impairment, as evidenced by a reduced long‐time rise in creatinine and slower eGFR decline, suggesting a potential protective effect on kidney function despite the initial mild increase in creatinine levels. Additionally, a nationwide population‐based cohort study in Korea found that fenofibrate use in patients taking statins was associated with a low risk of incident end‐stage renal disease. More studies are needed to confirm this effect.	77.63%

### 3.2 Statements

#### 3.2.1 Screening and Diagnosis

**Statement 1.1** This statement achieved 92.11% agreement. The expert panel recommends individualizing the frequency of eye screening based on type of diabetes, level of glycemic control, and retinopathy severity [13]. Existing guidelines recommend that patients with Type 1 diabetes should be offered an eye screening within 5 years of diagnosis, while patients with Type 2 diabetes should have an immediate screening [11, 14, 15]. This statement stresses that patients with uncontrolled Type 2 diabetes need more frequent screening as they have a higher risk of retinopathy progression [15]. It is also highlighted that counseling of patients is important to increase screening attendance [11].

**Statement 1.2** This statement achieved 86.84% agreement. There are many screening methods available for diabetic retinopathy, with dilated eye examination being the gold standard [14]. Retinal photography is also recommended to improve access to screening pathways; digital is preferred if cost is not a barrier, and this can be conducted by any trained healthcare provider [13].

**Statement 1.3** This statement achieved 93.42% agreement. The expert panel recommends prompt referral to an ophthalmologist at the first signs of moderate or worse NPDR, PDR, or any significant changes, to confirm the stage of diabetic retinopathy and allow timely intervention [13]. Primary care physicians should be educated about when referral to ophthalmology is required for their patients with diabetes [14]. Furthermore, healthcare systems should implement integrated, multidisciplinary care models that promote coordinated communication between primary care providers, endocrinologists, and ophthalmologists. Standardized referral protocols are needed to ensure timely ophthalmologic evaluation.

**Statement 1.4** This statement achieved 93.42% agreement. Early identification and systemic treatment of diabetic retinopathy allow for timely intervention before the disease advances to proliferative stages, where the risk of vision‐threatening complications is significantly higher [5, 16]. This is also important to prevent the need for invasive interventions such as panretinal laser photocoagulation or anti‐vascular endothelial growth factor (VEGF) intravitreal injections [5, 16]. Systemic treatment refers to therapies that act throughout the body, rather than directly in the eye, such as medications for blood glucose, blood pressure, and lipid control. Large clinical trials have demonstrated the efficacy of one such systemic treatment, fenofibrate, in slowing the progression of diabetic retinopathy and reducing the requirement for laser therapy. The FIELD study found that fenofibrate significantly reduced the need for first laser treatment for diabetic retinopathy [6]. The ACCORD Eye Study showed that combined therapy with fenofibrate and statins reduced the progression of diabetic retinopathy in patients with Type 2 diabetes [7]. Additionally, a recent meta‐analysis consolidates evidence from randomized controlled trials, confirming that fenofibrate therapy is associated with a lower incidence of laser treatment [16].

**Statement 1.5** This statement achieved 85.53% agreement. Reducing HbA1c significantly slows the onset and progression of diabetic retinopathy. The evidence supports an HbA1c target of < 7% for most adults, as recommended by clinical guidelines [11, 17, 18]. The expert panel recommends individualizing this target for each patient. This statement also stresses the need for a gradual decrease in patients with poor glycemic control (HbA1c ~ > 8.5%). Intensive glucose lowering in these patients can lead to short‐term worsening of diabetic retinopathy, a phenomenon particularly observed in patients initiating glucagon‐like peptide 1 receptor agonists (GLP‐1 RAs) and insulin treatments [15, 19]. The underlying mechanism of GLP‐1 RAs on diabetic retinopathy needs to be further investigated.

**Statement 1.6** This statement achieved 88.16% agreement. To enable effective screening for diabetic retinopathy, the expert panel recommends a comprehensive screening model, including a diabetes register and electronic health record systems, as well as clearly defined referral pathways [13]. Telemedicine can enhance reach in rural or underserved regions, and mobile screening units can help overcome transportation and geographic barriers [13]. However, these strategies represent ideal conditions. In many low‐ and middle‐income settings, full implementation may not be feasible due to infrastructure limitations, lack of trained personnel, or technological constraints. In such contexts, the pathway elements can be simplified while maintaining the core principles of timely identification and referral.

#### 3.2.2 Treatment and Prevention of Progression

**Statement 2.1** This statement achieved 86.84% agreement. Conventional treatments for diabetic retinopathy are often invasive, costly, and typically reserved for advanced stages of disease [20]. The expert panel highlights a clear need for long‐term, minimally invasive systemic therapies that can be introduced earlier to prevent disease progression and preserve vision.

**Statement 2.2** This statement achieved 78.95% agreement. Multiple large‐scale randomized trials, including the FIELD, ACCORD Eye, and LENS studies, have demonstrated that fenofibrate significantly reduces the risk of diabetic retinopathy progression and the need for laser treatment, especially in patients with Type 2 diabetes [6, 7, 21]. This benefit is most evident when fenofibrate is used alongside standard risk factor management, such as glycemic, blood pressure, and lipid control. More clinical trials are needed to confirm the effect in patients with Type 1 diabetes.

**Statement 2.3** This statement achieved 78.95% agreement. The expert panel recommends that fenofibrate treatment be initiated at the first signs of mild or moderate non‐proliferative retinopathy, in line with the National Institute for Health and Care Excellence (NICE) guidelines [11]. It is stressed that patient education should clearly communicate that fenofibrate is used in this context for its retinopathy‐preventing effects, not solely as a lipid‐lowering agent. As with all systemic interventions, comprehensive risk factor control remains essential.

**Statement 2.4** This statement achieved 85.53% agreement. Although statins are effective in reducing low‐density lipoprotein (LDL) cholesterol and are widely used to prevent cardiovascular complications in patients with diabetes, current evidence suggests that statin therapy does not significantly influence the progression of diabetic retinopathy. Meta‐analyses, cohort studies, and epidemiological data have shown no consistent association between statin use and a reduced risk of developing diabetic retinopathy [22, 23, 24].

**Statement 2.5** This statement achieved 85.53% agreement. Large, randomized trials such as the ACCORD Eye Study have shown a significant reduction in retinopathy progression in patients treated with fenofibrate monotherapy, even when lipid levels were well‐controlled [7]. The LENS study confirmed fenofibrate's effect on diabetic retinopathy progression when used as monotherapy [21]. Additional real‐world data indicate that adding fenofibrate to statin therapy also reduces the risk of progression [25, 26].

**Statement 2.6** This statement achieved 89.47% agreement. The expert panel agreed that fenofibrate offers the greatest benefit in patients with pre‐existing diabetic retinopathy. The ACCORD Eye Study demonstrated that fenofibrate reduced the progression of diabetic retinopathy by 40% in patients who had mild to moderate non‐proliferative retinopathy at baseline [7]. Similarly, the FIELD study showed a 31% relative reduction in the need for first laser treatment among those with pre‐existing retinopathy [6]. A 2023 Cochrane review confirmed these findings, concluding that fenofibrate provides substantial retinal protection, particularly in individuals already showing early signs of the disease [27].

**Statement 2.7** This statement achieved 78.95% agreement. Available evidence suggests that fenofibrate may protect against diabetic retinopathy through multiple molecular mechanisms, primarily by activating PPAR‐*α*. This activation downregulates inflammatory mediators and VEGF, both key drivers of retinal vascular damage in diabetes [28]. Fenofibrate also reduces oxidative stress by enhancing antioxidant enzyme expression and limiting reactive oxygen species, thereby preserving retinal cell viability and vascular stability [29]. These cellular effects help explain the clinical benefits observed.

#### 3.2.3 Treatment Duration

**Statement 3.1** This statement achieved 81.58% agreement. Two randomized trials reported significantly reduced rates of diabetic retinopathy progression compared to placebo over a median treatment duration of 4.0 years [7, 21]. In the ACCORD Eye Study, the rates of progression of diabetic retinopathy were 6.5% with fenofibrate for intensive dyslipidemia therapy, versus 10.2% with placebo (adjusted odds ratio, 0.60; 95% CI, 0.42–0.87; *p* = 0.006) [7]. In the LENS study, 22.7% of the fenofibrate group had progression, compared to 29.2% in the placebo group (hazard ratio, 0.73; 95% CI, 0.58–0.91; *p* = 0.006) [21]. These findings suggest a durable protective effect against vision‐threatening complications. Therefore, the expert panel recommends that fenofibrate be considered as a long‐term treatment. However, this statement highlights that further long‐term studies are needed to confirm sustained benefits.

**Statement 3.2** This statement achieved 80.26% agreement. The FIELD study, a large, randomized trial, demonstrated that fenofibrate significantly reduced the need for laser therapy in adults with Type 2 diabetes, compared to placebo (hazard ratio, 0.69; 95% CI, 0.56–0.84; *p* = 0.0002) [6]. The expert panel called for additional research to evaluate its effects in patients with Type 1 diabetes and to assess its impact on other ophthalmologic treatments beyond laser therapy.

**Statement 3.3** This statement achieved 84.21% agreement. In the ACCORD Follow‐On study (ACCORDION), the initial 4‐year use of fenofibrate in combination with simvastatin reduced the risk of diabetic retinopathy progression by 40%. However, this benefit was not sustained after treatment was stopped, as retinopathy progression rates equalized between the fenofibrate and placebo groups during the follow‐up period, demonstrating that continued treatment is necessary to maintain protective effects [30].

#### 3.2.4 Fenofibrate and Kidney Function

**Statement 4.1** This statement achieved 82.89% agreement. Although fenofibrate is commonly associated with a mild and reversible increase in serum creatinine, this rise is not indicative of kidney damage. Creatinine levels usually stabilize without further progression during continued treatment and return to baseline after discontinuation. These effects have been consistently observed across randomized trials and large‐scale studies, indicating that the creatinine rise is predictable, non‐progressive, and reversible [30, 31, 32, 33, 34]. It is important that physicians, including primary care providers, recognize that this transient rise in serum creatinine is not a sign of kidney damage to avoid unnecessary discontinuation of fenofibrate treatment.

**Statement 4.2** This statement achieved 77.63% agreement. The expert panel highlighted that the increase in serum creatinine observed with fenofibrate use is not fully understood but appears to result from multiple physiological mechanisms. Evidence suggests that the elevation is due to reduced creatinine clearance, despite a preserved glomerular filtration rate (GFR) measured by inulin, indicating that tubular secretion of creatinine may be affected rather than overall kidney function [30, 32]. Altered renal hemodynamics may also contribute, alongside potential increases in creatinine production from muscle metabolism [35, 36].

**Statement 4.3** This statement achieved 77.63% agreement. In the FIELD study, patients receiving fenofibrate experienced a smaller rise in plasma creatinine over 5 years compared to placebo, and the rate of estimated GFR (eGFR) decline was significantly slower (−0.14 vs. −0.32 mL/min/1.73 m^2^/year; *p* = 0.01), indicating a modest renoprotective effect despite the initial increase in creatinine [33]. Similarly, in the ACCORD study, fenofibrate‐treated participants had a significantly slower decline in eGFR (mean difference, +0.15 mL/min/1.73 m^2^/year; *p* < 0.001), and the early rise in creatinine was reversible upon discontinuation [37]. Additionally, a nationwide Korean cohort study found that among statin users, those who also received fenofibrate had a 24% lower risk of developing end‐stage renal disease (adjusted hazard ratio, 0.763; 95% CI, 0.710–0.821) compared to statin‐only users [38]. These findings support a potential protective role of fenofibrate on kidney function, though further studies are needed to confirm these effects across a broader population.

## 4 Discussion

This Delphi consensus represents a significant step toward harmonizing clinical approaches to the prevention of diabetic retinopathy progression, particularly through the use of fenofibrate as a systemic therapy. As diabetic retinopathy continues to pose a considerable public health burden, there is an urgent need to align clinical practices with the growing body of evidence supporting pharmacological prevention strategies. By engaging a diverse panel of international experts, this consensus provides a unified perspective on the role of endocrinologists in the screening, diagnosis, and treatment of diabetic retinopathy.

A major theme that arose from the consensus is the need for comprehensive, multidisciplinary models of care for diabetic retinopathy, integrating endocrinology, ophthalmology, and primary care. System‐level improvements, such as electronic health record integration, telemedicine screening, and clearly defined referral pathways, were highlighted as essential components for optimizing patient outcomes by facilitating early intervention. The expert panel recognized that while ideal care pathways may not be universally feasible, any improvement in screening and referral coordination can significantly impact the trajectory of disease progression.

A key outcome of the consensus is the strong endorsement of fenofibrate as a systemic therapy for Type 2 diabetes patients with early NPDR. While fenofibrate has been widely studied in trials such as ACCORD Eye, FIELD, and LENS, its integration into routine clinical practice has been inconsistent globally due to a lack of detailed guidelines. The panel's agreement that fenofibrate should be initiated at the first signs of mild or moderate NPDR reflects a critical paradigm shift, advocating for earlier, preventive intervention by endocrinologists rather than reactive treatment in advanced disease stages. This strategy may reduce the need for invasive interventions such as laser photocoagulation or intravitreal injections, which are often associated with higher costs and greater patient burden.

Importantly, the panel emphasized that fenofibrate's benefits extend beyond lipid control, which remains a commonly misunderstood aspect of its role in diabetic retinopathy management. The consensus reinforces the necessity for patient and provider education regarding fenofibrate's multifactorial effects, including anti‐inflammatory, anti‐angiogenic, and antioxidant mechanisms. These mechanisms underpin its retinal protective properties and support its use as a targeted diabetic retinopathy therapy rather than a general dyslipidemia treatment.

The expert panel also considered the importance of timing and duration of treatment. It was agreed that sustained treatment with fenofibrate is necessary to maintain its protective effect on the retina. Evidence from the ACCORDION follow‐up study showed that the benefits of fenofibrate diminish upon discontinuation, suggesting that continuous therapy is essential to prevent diabetic retinopathy progression. Further investigation is warranted to assess the efficacy of long‐term fenofibrate treatment beyond the median duration of its clinical trials (4.0 years).

This consensus also addressed concerns surrounding fenofibrate's impact on kidney function. While fenofibrate is known to cause mild and reversible elevations in serum creatinine, the expert panel agreed that this effect is not indicative of renal damage. Emerging data even suggest a potential renoprotective effect, although additional mechanistic and longitudinal studies are needed to confirm this benefit across diverse populations.

The strengths of this study include the rigorous Delphi methodology, which enabled systematic, iterative feedback and fostered convergence of expert opinion. The inclusion of both a core and an extended panel enhanced the representativeness and generalizability of the findings. The panelists were endocrinology experts from nine countries, reflecting the diabetic retinopathy treatment landscape around the world. Additionally, each statement is supported by a number of clinical trials and peer‐reviewed publications. Lastly, there was a high level of agreement, with 14 of the 19 statements achieving > 80% agreement.

In addition to the strengths, some limitations should be acknowledged. While the Delphi method is well‐suited for building expert consensus, it does not replace high‐quality empirical evidence. The recommendations, although informed by clinical trial data, remain opinion‐based and may not fully capture the heterogeneity of real‐world clinical practice. Lastly, although the process incorporated experts from multiple continents, certain regions were underrepresented, which may limit the applicability of the recommendations in these areas.

## 5 Conclusion

This Delphi consensus represents a meaningful advancement in the global approach to the prevention of diabetic retinopathy progression. It offers actionable, expert‐endorsed recommendations for endocrinologists that support the early identification of diabetic retinopathy and prompt intervention with fenofibrate. While further studies are required to address remaining evidence gaps, these findings lay the groundwork for more proactive and unified management strategies worldwide.

## Author Contributions

Richard C. O'Brien consulted on the initial statements, voted, commented, and agreed on the statements, approved the final consensus report, and reviewed and edited the manuscript. Khalifa Abdalla, Xuehong Dong, Mohamed Hassanein, Qiuhe Ji, Sakir Ozgur Keskek, Patricio Lopez‐Jaramillo, Roopa Mehta, Hussein Raef, and Kevin Tan voted, commented, and agreed on the statements, approved the final consensus report, and reviewed and edited the manuscript. All authors approved the final version of the manuscript.

## Funding

This study was funded by Abbott Products Operations AG, Switzerland.

## Ethics Statement

The authors have nothing to report.

## Conflicts of Interest

Richard C. O'Brien has received lecture and advisory board fees from Abbott, Amgen, AstraZeneca, Boehringer Ingelheim, Eli Lilly, MSD, Novartis, and Novo Nordisk. Patricio Lopez‐Jaramillo has received lecture fees from Abbott, Menarini, and Merck. Roopa Mehta has received lecture fees from Abbott, Adium, Amgen, AstraZeneca, Bayer, Boehringer Ingelheim, Eli Lilly, Novartis, Novenaries, Sanofi, and Silanes. Mohamed Hassanein has attended educational activities and/or advisory board meetings for Abbott, Bayer, Lilly, Novo Nordisk, Sanofi, Boehringer Ingelheim, Merck, MSD, and Medtronic. Khalifa Abdalla, Xuehong Dong, Qiuhe Ji, Sakir Ozgur Keskek, Hussein Raef, and Kevin Tan have declare no conflicts of interest.

## Supporting information


**Supplementary Table 1** Initial consensus statements and round 1 agreement level.

## Data Availability

The data that supports the findings of this study are available in the Supporting Information of this article.

## References

[1] GBD 2019 Blindness and Vision Impairment Collaborators; Vision Loss Expert Group of the Global Burden of Disease Study , “Causes of Blindness and Vision Impairment in 2020 and Trends Over 30 Years, and Prevalence of Avoidable Blindness in Relation to VISION 2020: The Right to Sight: An Analysis for the Global Burden of Disease Study [Published Correction Appears in Lancet Glob Health. 2021 Apr;9(4):e408.],” Lancet Global Health 9, no. 2 (2021): e144–e160.33275949

[2] H. Sun , P. Saeedi , S. Karuranga , et al., “IDF Diabetes Atlas: Global, Regional and Country‐Level Diabetes Prevalence Estimates for 2021 and Projections for 2045,” Diabetes Research and Clinical Practice 183 (2022): 109119. 34879977 10.1016/j.diabres.2021.109119 PMC11057359

[3] J. W. Yau , S. L. Rogers , R. Kawasaki , et al., “Global Prevalence and Major Risk Factors of Diabetic Retinopathy,” Diabetes Care 35, no. 3 (2012): 556–564. 22301125 10.2337/dc11-1909 PMC3322721

[4] C. P. Wilkinson , F. L. Ferris, 3rd , R. E. Klein , et al., “Proposed International Clinical Diabetic Retinopathy and Diabetic Macular Edema Disease Severity Scales,” Ophthalmology 110, no. 9 (2003): 1677–1682. 13129861 10.1016/S0161-6420(03)00475-5

[5] American Diabetes Association Professional Practice Committee , “12. Retinopathy, Neuropathy, and Foot Care: Standards of Medical Care in Diabetes—2024,” Diabetes Care 47, no. Suppl 1 (2024): S231–S243. 38078577 10.2337/dc24-S012 PMC10725803

[6] A. C. Keech , P. Mitchell , P. A. Summanen , et al., “Effect of Fenofibrate on the Need for Laser Treatment for Diabetic Retinopathy (FIELD Study): A Randomised Controlled Trial,” Lancet 370, no. 9600 (2007): 1687–1697. 17988728 10.1016/S0140-6736(07)61607-9

[7] ACCORD Study Group; ACCORD Eye Study Group , E. Y. Chew , W. T. Ambrosius , et al., “Effects of Medical Therapies on Retinopathy Progression in Type 2 Diabetes,” New England Journal of Medicine 363, no. 3 (2010): 233–244. 20587587 10.1056/NEJMoa1001288 PMC4026164

[8] E. Y. Chew , M. D. Davis , R. P. Danis , et al., “The Effects of Medical Management on the Progression of Diabetic Retinopathy in Persons With Type 2 Diabetes: The Action to Control Cardiovascular Risk in Diabetes (ACCORD) Eye Study,” Ophthalmology 121, no. 12 (2014): 2443–2451. 25172198 10.1016/j.ophtha.2014.07.019 PMC4252767

[9] D. Preiss , E. Spata , R. R. Holman , et al., “Effect of Fenofibrate Therapy on Laser Treatment for Diabetic Retinopathy: A Meta‐Analysis of Randomized Controlled Trials,” Diabetes Care 45, no. 1 (2022): e1–e2. 34876531 10.2337/dc21-1439 PMC8753761

[10] International Council of Ophthalmology (ICO) , “ICO Guidelines for Diabetic Eye Care,” 2013, https://icoph.org/eye‐care‐delivery/diabetic‐eye‐care/.

[11] National Institute for Health and Care Excellence (NICE) , “Type 2 Diabetes in Adults: Management,” NG28, 2015, https://www.nice.org.uk/guidance/NG28. 26741015

[12] The Royal Australian College of General Practitioners (RACGP) , “Management of Type 2 Diabetes: A Handbook for General Practice,” 2024, https://www.racgp.org.au/clinical‐resources/clinical‐guidelines/key‐racgp‐guidelines/view‐all‐racgp‐guidelines/management‐of‐type‐2‐diabetes/introduction.

[13] WHO Regional Office for Europe , “Diabetic Retinopathy Screening: A Short Guide. Increase Effectiveness, Maximize Benefits and Minimize Harm,” 2021, https://www.who.int/europe/publications/i/item/9789289055321.

[14] American Academy of Ophthalmology Preferred Practice Pattern Retina/Vitreous Committee , “Diabetic Retinopathy Preferred Practice Pattern,” 2019, https://www.aao.org/education/preferred‐practice‐pattern/diabetic‐retinopathy‐ppp.

[15] M. A. Bethel , R. Diaz , N. Castellana , I. Bhattacharya , H. C. Gerstein , and M. C. Lakshmanan , “HbA_1c_ Change and Diabetic Retinopathy During GLP‐1 Receptor Agonist Cardiovascular Outcome Trials: A Meta‐Analysis and Meta‐Regression,” Diabetes Care 44, no. 1 (2021): 290–296. 33444163 10.2337/dc20-1815 PMC7783944

[16] G. Lingam and T. Y. Wong , “Systemic Medical Management of Diabetic Retinopathy,” Middle East African Journal of Ophthalmology 20, no. 4 (2013): 301–308. 24339679 10.4103/0974-9233.120010 PMC3841947

[17] S. Genuth , R. Eastman , R. Kahn , et al., “American Diabetes Association. Implications of the United Kingdom Prospective Diabetes Study,” Diabetes Care 26, no. Suppl 1 (2003): S28–S32. 12502617 10.2337/diacare.26.2007.s28

[18] M. J. Davies , V. R. Aroda , B. S. Collins , et al., “Management of Hyperglycemia in Type 2 Diabetes, 2022. A Consensus Report by the American Diabetes Association (ADA) and the European Association for the Study of Diabetes (EASD),” Diabetes Care 45, no. 11 (2022): 2753–2786. 36148880 10.2337/dci22-0034 PMC10008140

[19] The Diabetes Control and Complications Trial Research Group , “Early Worsening of Diabetic Retinopathy in the Diabetes Control and Complications Trial,” Archives of Ophthalmology 116, no. 7 (1998): 874–886, Erratum in: Archives of Ophthalmology (1998) 116, no. 11:1469. 9682700 10.1001/archopht.116.7.874

[20] R. Simó , S. Ballarini , J. Cunha‐Vaz , et al., “Non‐Traditional Systemic Treatments for Diabetic Retinopathy: An Evidence‐Based Review,” Current Medicinal Chemistry 22, no. 21 (2015): 2580–2589. 25989912 10.2174/0929867322666150520095923 PMC4997935

[21] D. Preiss , J. Logue , E. Sammons , et al., “Effect of Fenofibrate on Progression of Diabetic Retinopathy,” NEJM Evidence 3, no. 8 (2024): EVIDoa2400179. 38905569 10.1056/EVIDoa2400179 PMC7616293

[22] V. Mozetic , R. L. Pacheco , C. O. C. Latorraca , and R. Riera , “Statins and/or Fibrates for Diabetic Retinopathy: A Systematic Review and Meta‐Analysis,” Diabetology & Metabolic Syndrome 11 (2019): 92. 31719846 10.1186/s13098-019-0488-9 PMC6839185

[23] E. Meer , J. C. Bavinger , Y. Yu , P. Hua , B. McGeehan , and B. L. VanderBeek , “Statin Use and the Risk of Progression to Vision Threatening Diabetic Retinopathy,” Pharmacoepidemiology and Drug Safety 31, no. 6 (2022): 652–660. 35253307 10.1002/pds.5426 PMC10210055

[24] J. Zhang and G. McGwin, Jr. , “Association of Statin Use With the Risk of Developing Diabetic Retinopathy,” Archives of Ophthalmology 125, no. 8 (2007): 1096–1099. 17698757 10.1001/archopht.125.8.1096

[25] N. H. Kim , J. Choi , Y. H. Kim , H. Lee , and S. G. Kim , “Addition of Fenofibrate to Statins Is Associated With Risk Reduction of Diabetic Retinopathy Progression in Patients With Type 2 Diabetes and Metabolic Syndrome: A Propensity‐Matched Cohort Study,” Diabetes & Metabolism 49, no. 3 (2023): 101428. 36720383 10.1016/j.diabet.2023.101428

[26] M. P. Hermans , “Impact of Fenofibrate on Type 2 Diabetes Patients With Features of the Metabolic Syndrome: Subgroup Analysis From FIELD,” Current Cardiology Reviews 6, no. 2 (2010): 112–118. 21532777 10.2174/157340310791162686 PMC2892076

[27] S. Y. Kataoka , N. Lois , S. Kawano , Y. Kataoka , K. Inoue , and N. Watanabe , “Fenofibrate for Diabetic Retinopathy,” Cochrane Database of Systematic Reviews 6, no. 6 (2023): CD013318. 37310870 10.1002/14651858.CD013318.pub2 PMC10264082

[28] Y. Chen , Y. Hu , M. Lin , et al., “Therapeutic Effects of PPARα Agonists on Diabetic Retinopathy in Type 1 Diabetes Models,” Diabetes 62, no. 1 (2013): 261–272. 23043158 10.2337/db11-0413 PMC3526044

[29] Y. J. Hsu , C. W. Lin , S. L. Cho , W. S. Yang , C. M. Yang , and C. H. Yang , “Protective Effect of Fenofibrate on Oxidative Stress‐Induced Apoptosis in Retinal‐Choroidal Vascular Endothelial Cells: Implication for Diabetic Retinopathy Treatment,” Antioxidants 9, no. 8 (2020): 712. 32764528 10.3390/antiox9080712 PMC7464418

[30] Action to Control Cardiovascular Risk in Diabetes Follow‐On (ACCORDION) Eye Study Group and the Action to Control Cardiovascular Risk in Diabetes Follow‐On (ACCORDION) Study Group , “Persistent Effects of Intensive Glycemic Control on Retinopathy in Type 2 Diabetes in the Action to Control Cardiovascular Risk in Diabetes (ACCORD) Follow‐On Study,” Diabetes Care 39, no. 7 (2016): 1089–1100. 27289122 10.2337/dc16-0024 PMC4915557

[31] J. C. Ansquer , R. N. Dalton , E. Caussé , D. Crimet , K. Le Malicot , and C. Foucher , “Effect of Fenofibrate on Kidney Function: A 6‐Week Randomized Crossover Trial in Healthy People,” American Journal of Kidney Diseases 51, no. 6 (2008): 904–913. 18501783 10.1053/j.ajkd.2008.01.014

[32] T. M. Davis , R. Ting , J. D. Best , et al., “Effects of Fenofibrate on Renal Function in Patients With Type 2 Diabetes Mellitus: The Fenofibrate Intervention and Event Lowering in Diabetes (FIELD) Study,” Diabetologia 54, no. 2 (2011): 280–290. 21052978 10.1007/s00125-010-1951-1

[33] A. Hadjivasilis , P. Kouis , A. Kousios , and A. Panayiotou , “The Effect of Fibrates on Kidney Function and Chronic Kidney Disease Progression: A Systematic Review and Meta‐Analysis of Randomised Studies,” Journal of Clinical Medicine 11, no. 3 (2022): 768. 35160220 10.3390/jcm11030768 PMC8836930

[34] ACCORD Study Group , H. N. Ginsberg , M. B. Elam , et al., “Effects of Combination Lipid Therapy in Type 2 Diabetes Mellitus,” New England Journal of Medicine 362, no. 17 (2010): 1563–1574. 20228404 10.1056/NEJMoa1001282 PMC2879499

[35] C. Hottelart , N. el Esper , J. M. Achard , A. Pruna , and A. Fournier , “Le fénofibrate augmente la créatininémie mais n'altère pas le débit de filtration glomérulaire chez les patients présentant une insuffisance rénale modérée [Fenofibrate Increases Blood Creatinine, But Does Not Change the Glomerular Filtration Rate in Patients With Mild Renal Insufficiency],” Néphrologie 20, no. 1 (1999): 41–44. 10081035

[36] C. R. McQuade , J. Griego , J. Anderson , and A. B. Pai , “Elevated Serum Creatinine Levels Associated With Fenofibrate Therapy,” American Journal of Health‐System Pharmacy 65, no. 2 (2008): 138–141. 18192258 10.2146/ajhp070005

[37] J. C. Mychaleckyj , T. Craven , U. Nayak , et al., “Reversibility of Fenofibrate Therapy‐Induced Renal Function Impairment in ACCORD Type 2 Diabetic Participants,” Diabetes Care 35, no. 5 (2012): 1008–1014. 22432114 10.2337/dc11-1811 PMC3329840

[38] Y. Y. Hyun , K. S. Kim , S. Hong , K. Han , and C. Y. Park , “Fenofibrate and Risk of End‐Stage Renal Disease: A Nationwide Cohort Study,” Diabetes, Obesity & Metabolism 26, no. 10 (2024): 4583–4590. 10.1111/dom.15815 39075919

