# Supplementary material for: Delphi Consensus on the Use of Fenofibrate as Systemic Therapy for the Prevention of Diabetic Retinopathy Progression
Source: J Diabetes. 2026 Feb 12;18(2):e70193. doi: 10.1111/1753-0407.70193 (PMC12895469; doi:10.1111/1753-0407.70193)
Supplement: Supplementary file 1 — Supplementary Table 1 Initial consensus statements and round 1 agreement level. [file JDB-18-e70193-s001.docx]

*Supplementary Table 1 Initial consensus statements and round 1 agreement level*

| Statement Number | Statement Content | Agreement Level |
| --- | --- | --- |
| 1.1 | Adults with type 1 diabetes should be offered annual eye screenings beginning 5 years after diagnosis. Adults with type 2 diabetes should be offered a prompt eye screening at the time of diagnosis and annually thereafter. The aim is to identify signs of diabetic retinopathy and facilitate earlier intervention before the disease progresses to be vision threatening. Patients should be counseled on the importance of attending yearly screening appointments, even if they are asymptomatic, to reduce their risk of diabetes-associated vision impairment. | 97.50% |
| 1.2 | Screening for diabetic retinopathy should utilize dilated eye examination and retinal photography as primary methods. The screening should be conducted by an optometrist or ophthalmologist. | 57.50% |
| 1.3 | Any patient identified with evidence of moderate or worse non-proliferative diabetic retinopathy or proliferative diabetic retinopathy should be promptly referred to an ophthalmologist for further management. | 95.00% |
| 1.4 | Early identification and subsequent systemic treatment of diabetic retinopathy is essential to prevent progression to proliferative retinopathy and avoid costly, invasive, and high-risk treatments for advanced disease, such as retinal laser and intravitreal injections. | 97.50% |
| 1.5 | Strict glycemic control should be emphasized as a critical measure to prevent the progression of diabetic retinopathy, alongside management of other systemic risk factors like hypertension and lipid levels. A HbA1c target of <7% is reasonable for most adults, but the target should be personalized based on disease duration, life expectancy, use of pharmacological agents, comorbidities, and established vascular complications. | 97.50% |
| 1.6 | Effective screening for diabetic retinopathy requires a systematized screening pathway to be in place in order for patients to receive preventative treatment in a timely manner. This should include:   - Electronic Health Record (EHR)-based tracking systems to send automated invitations and reminders and to flag patients who are overdue for follow-up appointments - Streamlined referral pathways, with direct electronic communication between primary care providers and specialists, prompt follow-up, and clear guidance for patients on the next steps - The use of telemedicine to maintain regular contact with patients, especially those in rural or underserved areas, to reduce the likelihood of missed appointments or delays in follow-up | 82.50% |
| 2.1 | Most treatments that have conventionally been used to treat diabetic retinopathy are used to help halt disease progression or reduce vision loss at fairly advanced stages and are invasive. Systemic treatment options that are minimally invasive are needed to prevent disease progression at the early stages and help preserve vision. | 92.50% |
| 2.2 | Fenofibrate offers an effective therapy to slow the progression of diabetic retinopathy and reduce the need for more expensive and invasive treatments. | 85.00% |
| 2.3 | Physicians should prescribe fenofibrate at the first signs of mild or moderate non-proliferative retinopathy, even if blood glucose is well controlled, to prevent the progression to proliferative diabetic retinopathy (PDR). | 80.00% |
| 2.4 | Treatment with statin medications, which predominantly addresses dyslipidemia through the reduction of low-density lipoprotein (LDL), does not appear to significantly alter the course of diabetic retinopathy. | 82.50% |
| 2.5 | Fenofibrate has been shown to reduce the progression of existing diabetic retinopathy when used as monotherapy and also in combination with a statin. | 87.50% |
| 2.6 | Fenofibrate treatment is particularly beneficial for patients with pre-existing diabetic retinopathy. Studies show that its use provides significant protection against further retinal damage, particularly in those already exhibiting early signs of the disease. | 90.00% |
| 3.1 | Fenofibrate treatment should be considered indefinite for patients with non-proliferative and proliferative diabetic retinopathy as it may slow disease progression and protect against vision-threatening complications. | 67.50% |
| 3.2 | Long-term treatment with fenofibrate can reduce the need for more invasive ophthalmologic interventions, such as laser therapy or anti-VEGF injections. | 82.50% |
| 3.3 | The therapeutic benefits of fenofibrate are lost once treatment is discontinued, and therefore sustained use is crucial to maintain its protective effects. | 87.50% |
| 4.1 | When fenofibrate is initiated, a mild and reversible increase in serum creatinine levels is commonly observed, typically within the first few weeks of treatment. After the initial rise, creatinine levels usually stabilize and remain elevated but do not increase further throughout the duration of fenofibrate treatment. Upon discontinuation of fenofibrate, creatinine levels typically return to baseline or near-baseline values. | 92.50% |
| 4.2 | The mechanism behind the increase in serum creatinine levels is not yet fully understood, but it appears to be partly due to reduced creatinine clearance, without reduced inulin-derived glomerular filtration rate (GFR), indicating a potential interference with the active secretion pathway of creatinine in the proximal tube, rather than altered glomerular function. Altered renal hemodynamics may also play a causative role. | 82.50% |
| 4.3 | Treatment with fenofibrate has been shown to slow the progression of renal function impairment, as evidenced by a reduced long-time rise in creatinine and slower eGFR decline, suggesting a protective effect on kidney function despite the initial mild increase in creatinine levels. | 75.00% |
